# Supplementary material for: Hot spot prediction in protein-protein interactions by an ensemble system
Source: BMC Syst Biol. 2018 Dec 31;12(Suppl 9):132. doi: 10.1186/s12918-018-0665-8 (PMC6311905; doi:10.1186/s12918-018-0665-8)
Supplement: Supplementary file 2 — All relevant MATLAB code. A simple Matlab implement of our predictor. (ZIP 19,742 kb) [file 12918_2018_665_MOESM2_ESM.zip › Additional file 2/Read ASA/ASA Property/SKEMPI/2NOJB.txt]

 NetSurfP - Protein Surface Accessibility and Secondary Structure Predictions

  

|  |  |  |
| --- | --- | --- |
|  |  | NetSurfP - Protein Surface Accessibility and Secondary Structure Predictions  Technical University of Denmark |

  


---

```
# For publication of results, please cite:
# A generic method for assignment of reliability scores applied to solvent accessibility predictions.
# Bent Petersen, Thomas Nordahl Petersen, Pernille Andersen, Morten Nielsen and Claus Lundegaard
# BMC Structural Biology 2009, 9:51 doi:10.1186/1472-6807-9-51
#
# Column 1: Class assignment - B for buried or E for Exposed - Threshold: 25% exposure, but not based on RSA
# Column 2: Amino acid
# Column 3: Sequence name
# Column 4: Amino acid number
# Column 5: Relative Surface Accessibility - RSA
# Column 6: Absolute Surface Accessibility
# Column 7: Z-fit score for RSA prediction
# Column 8: Probability for Alpha-Helix
# Column 9: Probability for Beta-strand
# Column 10: Probability for Coil
E N  2NOJB                  1    0.940 137.572   0.812   0.003   0.003   0.994
E K  2NOJB                  2    0.628 129.221   0.760   0.430   0.016   0.555
E K  2NOJB                  3    0.567 116.735   0.888   0.660   0.049   0.291
E V  2NOJB                  4    0.265  40.700   0.153   0.751   0.050   0.199
E V  2NOJB                  5    0.278  42.698   0.064   0.751   0.050   0.199
E D  2NOJB                  6    0.518  74.658   0.993   0.751   0.050   0.199
B A  2NOJB                  7    0.115  12.717  -1.079   0.879   0.010   0.111
E Q  2NOJB                  8    0.433  77.334   0.497   0.923   0.002   0.076
E K  2NOJB                  9    0.517 106.367   0.825   0.970   0.001   0.030
B A  2NOJB                 10    0.086   9.466   0.235   0.970   0.001   0.030
B V  2NOJB                 11    0.093  14.217   0.194   0.970   0.001   0.030
E E  2NOJB                 12    0.564  98.548   1.118   0.970   0.001   0.030
E L  2NOJB                 13    0.263  48.100   0.764   0.923   0.002   0.076
B F  2NOJB                 14    0.070  14.149  -0.439   0.858   0.002   0.139
E K  2NOJB                 15    0.556 114.266   0.866   0.879   0.010   0.111
E R  2NOJB                 16    0.572 130.988   0.500   0.802   0.014   0.185
B T  2NOJB                 17    0.282  39.100  -0.404   0.561   0.047   0.393
E R  2NOJB                 18    0.383  87.799   0.111   0.386   0.097   0.517
E T  2NOJB                 19    0.417  57.782   0.092   0.321   0.252   0.427
B V  2NOJB                 20    0.181  27.866   0.157   0.578   0.229   0.194
E A  2NOJB                 21    0.396  43.661  -0.570   0.605   0.105   0.290
E T  2NOJB                 22    0.387  53.691  -0.387   0.660   0.049   0.291
B H  2NOJB                 23    0.304  55.316  -0.340   0.751   0.050   0.199
E R  2NOJB                 24    0.441 101.058   0.366   0.751   0.050   0.199
E K  2NOJB                 25    0.535 110.152   0.706   0.751   0.050   0.199
B A  2NOJB                 26    0.174  19.208  -1.111   0.660   0.049   0.291
E Q  2NOJB                 27    0.458  81.727   0.059   0.561   0.047   0.393
E R  2NOJB                 28    0.506 115.966   0.732   0.538   0.173   0.289
B A  2NOJB                 29    0.185  20.387  -0.605   0.453   0.248   0.299
B V  2NOJB                 30    0.140  21.503   0.353   0.252   0.423   0.325
E N  2NOJB                 31    0.371  54.329  -0.184   0.169   0.612   0.219
B L  2NOJB                 32    0.157  28.765   0.248   0.169   0.612   0.219
B I  2NOJB                 33    0.147  27.287   0.145   0.169   0.612   0.219
B H  2NOJB                 34    0.308  55.952  -0.284   0.169   0.612   0.219
B F  2NOJB                 35    0.164  32.814   0.050   0.268   0.505   0.227
E Q  2NOJB                 36    0.469  83.746   0.363   0.352   0.332   0.316
E H  2NOJB                 37    0.356  64.793  -0.056   0.453   0.248   0.299
E S  2NOJB                 38    0.465  54.510   0.287   0.538   0.173   0.289
B Y  2NOJB                 39    0.260  55.455   0.160   0.605   0.105   0.290
E E  2NOJB                 40    0.591 103.283   0.348   0.751   0.050   0.199
E K  2NOJB                 41    0.502 103.364   0.288   0.802   0.014   0.185
E K  2NOJB                 42    0.486  99.888   0.695   0.879   0.010   0.111
E K  2NOJB                 43    0.421  86.579   1.007   0.938   0.007   0.055
B L  2NOJB                 44    0.124  22.631   0.108   0.938   0.007   0.055
E Q  2NOJB                 45    0.428  76.441   0.634   0.938   0.007   0.055
E R  2NOJB                 46    0.586 134.263   0.552   0.879   0.010   0.111
E Q  2NOJB                 47    0.394  70.422   0.636   0.911   0.033   0.057
B I  2NOJB                 48    0.062  11.544   0.553   0.802   0.014   0.185
E D  2NOJB                 49    0.504  72.598   0.113   0.751   0.050   0.199
B L  2NOJB                 50    0.289  52.934   0.120   0.701   0.107   0.192
B V  2NOJB                 51    0.114  17.460  -0.158   0.649   0.163   0.188
B L  2NOJB                 52    0.204  37.371   0.195   0.538   0.173   0.289
E K  2NOJB                 53    0.548 112.785   0.853   0.428   0.171   0.402
E Y  2NOJB                 54    0.289  61.717   0.235   0.307   0.165   0.527
E N  2NOJB                 55    0.525  76.919  -0.612   0.199   0.152   0.649
E T  2NOJB                 56    0.545  75.522  -0.191   0.118   0.150   0.732
E L  2NOJB                 57    0.354  64.854  -0.843   0.052   0.084   0.864
E K  2NOJB                 58    0.850 174.763   0.949   0.003   0.003   0.994

Explain the output.  Go back.
```

---
